# Derepression of Cancer/Testis Antigens in cancer is associated with distinct patterns of DNA Hypomethylation

**DOI:** 10.1186/1471-2407-13-144

**Published:** 2013-03-22

**Authors:** Robert Kim, Prakash Kulkarni, Sridhar Hannenhalli

**Affiliations:** 1 James Buchanan Brady Urological Institute, The Johns Hopkins University School of Medicine, 600 North Wolfe Street, Baltimore, MD, 21287, USA; 2 Department of Cell Biology & Molecular Genetics, and Center for Bioinformatics & Computational Biology, University of Maryland, 3104G Biomolecular Sciences Building (#296), College Park, MD, USA; 3 Present address: Department of Neurology, Yale University School of Medicine, New Haven, CT, USA

**Keywords:** DNA hypomethylation, Cancer/Testis antigens, Lamina attachment domains, Insulator regions

## Abstract

**Background:**

The Cancer/Testis Antigens (CTAs) are a heterogeneous group of proteins whose expression is typically restricted to the testis. However, they are aberrantly expressed in most cancers that have been examined to date. Broadly speaking, the CTAs can be divided into two groups: the CTX antigens that are encoded by the X-linked genes and the non-X CT antigens that are encoded by the autosomes. Unlike the non-X CTAs, the CTX antigens form clusters of closely related gene families and their expression is frequently associated with advanced disease with poorer prognosis. Regardless however, the mechanism(s) underlying their selective derepression and stage-specific expression in cancer remain poorly understood, although promoter DNA demethylation is believed to be the major driver.

**Methods:**

Here, we report a systematic analysis of DNA methylation profiling data from various tissue types to elucidate the mechanism underlying the derepression of the CTAs in cancer. We analyzed the methylation profiles of 501 samples including sperm, several cancer types, and their corresponding normal somatic tissue types.

**Results:**

We found strong evidence for specific DNA hypomethylation of CTA promoters in the testis and cancer cells but not in their normal somatic counterparts. We also found that hypomethylation was clustered on the genome into domains that coincided with nuclear lamina-associated domains (LADs) and that these regions appeared to be insulated by CTCF sites. Interestingly, we did not observe any significant differences in the hypomethylation pattern between the CTAs without CpG islands and the CTAs with CpG islands in the proximal promoter.

**Conclusion:**

Our results corroborate that widespread DNA hypomethylation appears to be the driver in the derepression of CTA expression in cancer and furthermore, demonstrate that these hypomethylated domains are associated with the nuclear lamina-associated domains (LADS). Taken together, our results suggest that wide-spread methylation changes in cancer are linked to derepression of germ-line-specific genes that is orchestrated by the three dimensional organization of the cancer genome.

## Background

The Cancer/Testis Antigens (CTAs) are a group of tumor-associated proteins that are typically expressed in normal male germ cells but are silent in normal somatic cells. However, they are aberrantly expressed in several types of cancers [1,2]. Because of this unique expression pattern, the CTAs are considered attractive targets for cancer biomarkers and immunotherapy [3].

Broadly speaking, the CTAs can be divided into two groups: the CTX antigens that are encoded by the X chromosome and the non-X CT antigens that are encoded by the autosomes. To date, 228 CTAs have been identified of which 120 CTAs (52%) map to the X chromosome (the CTX antigens) while the remaining (non-X CT antigens), are distributed on the 22 autosomes and the Y chromosome [4]. Interestingly, while some autosomes that are gene-poor such as chromosome 21 (only 425 genes), are enriched for CTA genes (1.6 CTAs/100 genes), others, that are gene-rich, such as chromosome 1 (3380 genes) and 7 (1764 genes), are very CTA-poor with only 0.3 CTAs and 0.06 CTAs/100 genes, respectively. However, among the sex chromosomes, while only 1 CTA is present on the Y chromosome, there are 7.5 CTAs/100 genes on the X chromosome – a 125-fold increase over chromosome 7 [4].

Furthermore, the CTX antigens are comprised of large gene families of closely related members and are frequently associated with advanced disease with poorer prognosis [5-10]. It is remarkable that as much as 10% of the genes on the X chromosome are estimated to belong to CT-X families [11]. Although the role of many of these tumor-associated antigens in the disease process remains unclear, emerging evidence indicates that they appear to function in several important cellular processes such as transcriptional regulation, signal transduction, and cell growth [3]. Some also appear to function as putative proto-oncogenes [12,13] and are associated with maintaining the undifferentiated state of stem cells [14-17].

More recently, a majority of the CTAs, especially the CTX antigens, were predicted to be intrinsically disordered proteins or IDPs [4]. IDPs are proteins that lack a rigid structure at least *in vitro*. Despite the lack of structure, most IDPs can transition from disorder to order upon binding to biological targets and often promote highly promiscuous interactions. Thus, IDPs play important roles in transcriptional regulation and signaling via regulatory protein networks and are frequently over-expressed in pathological conditions such as cancer [18,19]. Consistent with these observations, several CTAs are predicted to bind to DNA and their forced expression appears to increase cell growth implying a potential dosage-sensitive function [4]. Taken together, these observations provide a novel perspective on the CTAs implicating them in processing and transducing information in altered physiological states in a dosage-sensitive manner. Thus, understanding how the CTAs are selectively derepressed in cancer is an important question in cancer biology.

Although the mechanism promoting their derepression is not entirely clear, it is widely held that DNA methylation is one of the central mechanisms responsible for gene silencing [20-22]. For example, De Smet et al. have observed selective and genome-wide hypomethylation of MAGE-A1, one of the most studied CTAs in cancer cells, coincided with its activation [23-25]. Several other studies have also reported a similar trend in other CTA genes [26-30]. Roman-Gomez et al. discovered direct correlation between the methylation levels of the HAGE gene and its expression in myeloid leukemia [29]. Similarly, Cho et al. observed expression of the CAGE gene and its promoter hypomethylation in gastric cancer [27]. Yegnasubramanian et al. found that although the CT-X antigens undergo DNA hypomethylation and overexpression in primary prostate cancers, these changes were more pronounced in metastatic disease when many CT-X antigens were highly upregulated coincident with poorer prognosis [30]. Consistent with this hypothesis, other studies have shown that inhibiting DNA methyltransferase (DNMT) activity with 5 aza-deoxycytidine (5 AZA) results in robust somatic expression of a set of CTAs both *in vitro* and *in vivo*[31]. However, only a few studies have experimentally confirmed promoter demethylation following DNMT inhibition by 5AZA or silencing by siRNA [13,32] and in many cases CTA genes that lack CpG dinucleotides respond to DNMT inhibition while in other cases, despite the presence of CpG dinucleotides, the CTA genes are not derepressed. For instance, the *SPANX* genes, which lack a CpG island in the promoter region [33], respond robustly to 5 AZA treatments [34] implicating an indirect mechanisms underlying the response, although the presence of such sites at distal regions or within introns cannot be ruled out. It is therefore unclear to what extent these effects are mediated directly by promoter demethylation of the target genes as opposed to being indirectly driven by demethylation in conjunction with transcription factors that activate CTA expression.

Thus, it is obvious that our understanding of CTA gene regulation and mechanisms underlying their abrupt derepression in cancer has been not subjected to a genome-wide analysis to assess their generality. Such a genome-wide analysis has recently become possible due to availability of genome-scale methylation arrays and other related technologies. Here, using genome-scale methylation profiles of promoter CpG methylation in 501 samples that included 305 normal sperm and somatic cells, and 196 cancers, we employed a new metric to identify gene promoters that follow an expected pattern of CTA promoter methylation, i.e., promoters that are unmethylated in sperm, methylated in somatic tissues, but unmethylated in cancer tissues. The higher the metric value for a gene promoter, the more closely it follows the prototypical methylation pattern (PMP). Our analysis confirmed that CTA gene promoters broadly follow a PMP. At the genome level, we observed that PMP promoters tend to cluster together on the genome and the CTA genes appeared to strongly associate with such clusters. Furthermore, we discovered that the binding sites for CTCF, the generic ‘insulator protein’, demarcate the regions of PMP. Genomic regions with PMPs have been observed to be enriched for genes involved in defense response, immune response, and cytokine-cytokine receptor interaction [35,36]. We also found that a large fraction of CTAs genes, especially the ones associated with clusters of PMPs coincided with the nuclear lamina-associated domains (LADs). However, we did not observe any significant differences in the above hypomethylation patterns between the promoters with CpG islands (CGI) and promoters without CpG islands (non-CGI). Taken together, our results indicate that PMP is a broad phenomenon covering CTAs and that their derepression is significantly explained by previously observed broad domains of hypomethylation in cancer that are associated with LADs.

## Methods

### Methylation data

DNA methylation profiling data were obtained from the Gene Expression Omnibus (GEO) database [37]. To be consistent, methylation profile datasets from only one platform, Illumina HumanMethylation27 BeadChip (GPL8490) containing 27578 genome-wide promoter CG dinucleotides, was used in this study. Our methylation dataset contained 501 samples from profiling studies for five different tissues and conditions: breast cancer tissues (GSE26990), colorectal cancer tissues (GSE25062, GSE17648), normal sperm (GSE26974), and prostate cancer tissues (GSE26126). The processed and normalized data was used as provided. The CG loci were partitioned into two groups based on whether it belonged to a CpG islands (CGIs; 16561 loci) or not (non-CGIs; 11017 loci). Human CGIs were obtained from the UCSC Genome Browser (genome.ucsc.edu; Build 36, hg18).

### Estimating delta-PMP for a CTCF site

For each CTCF location, three nearest genes (by their transcription start sites) 5’ of the CTCF site and three nearest genes 3’ of the CTCF site were determined using ENSEMBL gene annotation (http://www.ensembl.org). A CTCF site was included for the analysis, if at least one of the three promoters to the left of the CTCF site and at least one of the three promoters to the right of the site had a CG locus represented in the methylation dataset. We computed the average PMP-sim for the promoters to the left and average PMP-sim for the promoters to the right. Then, we computed the absolute difference of those two averages as delta-PMP. The above procedure was performed separately for CGI loci and non-CGI loci.

## Results

### Method overview

**Figure 1:**
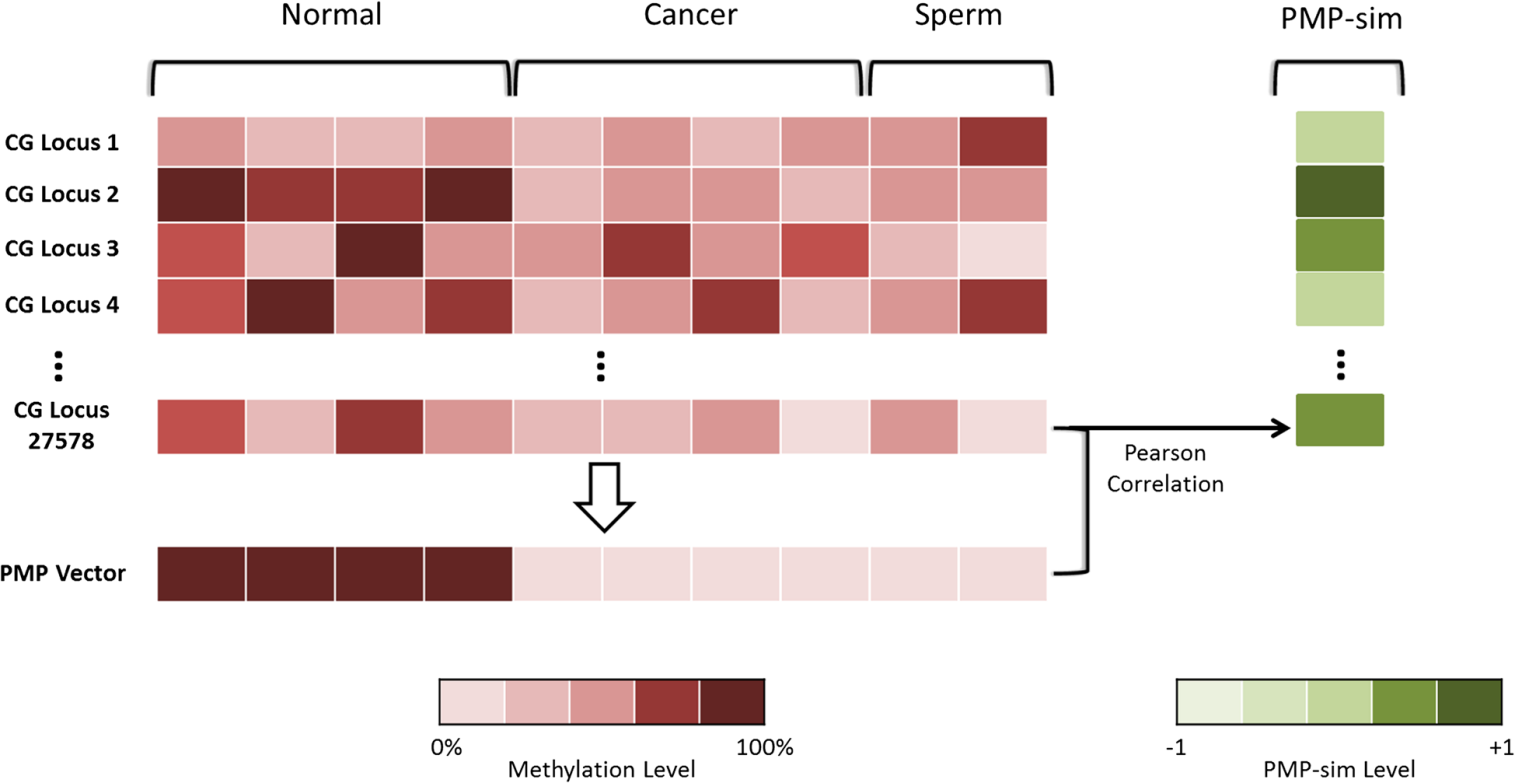
**Scheme to determine prototypical methylation pattern (PMP) and PMP-sim. **PMP is a vector or length 501 corresponding to 501 samples. For each sample, if it is cancer or sperm sample, the corresponding PMP vector element is assigned the minimum methylation value among all 27578 CG loci for that sample, and if the sample is from normal somatic tissue cancer the corresponding PMP vector element is assigned the maximum methylation value among all 27578 CG loci for that sample. For any CG locus, given its methylation pattern across 501 samples, the Pearson correlation between the methylation pattern and the PMP-vector is used to estimate PMP-sim for the CG locus.

Table 1 provides a summary of the Gene Expression Omnibus (GEO) datasets used in this study. Overall, the datasets included 501 distinct samples across 27578 CpG loci in the genome based on the HumanMethylation27 BeadChip data (Illumina, CA). Out of 501 samples, 289 (58%) of them were from tumors, while 212 (42%) were normal. Thus, the data is summarized as a matrix with 27578 rows and 501 columns with methylation intensity. As shown in Figure 1, we defined a prototypical methylation pattern (PMP) vector with 501 entries, one per sample, as follows. For each sample *i*, (1) if the sample was obtained from either normal sperm cells or a cancer tissue, then the minimum methylation value of all the genes for that sample was assigned to the *i*^*th*^ entry in PMP vector, and (2) if the sample was obtained from a normal somatic tissue, then the maximum methylation value of all the genes for that sample was assigned to the *i*^*th*^ entry in PMP vector. The PMP vector values are reported in Additional file 1: Table S1.

**Table 1:** GEO methylation studies included for analysis

**GEO accession**	**Tissue type**	**Total samples**	**Normal samples**	**Tumor samples**
GSE26990	Breast**	89	42	47
GSE26126	Prostate	193	98	95
GSE17648	Colorectal	44	22	22
GSE25062	Colorectal	154	29	125
GSE26974	Sperm	21	21	0

All methylation profiling was done based on Illumina HumanMethylation27 BeadChip (GEO platform GPL8490).

** Also included nine normal samples from other tissues (blood, spleen, colon, fetal brain, liver, testis, embryo, and adult brain).

To quantify how well a particular CpG location *j *conforms to the PMP, we computed the Pearson correlation between the *j*^*th *^row and the PMP-vector; we refer to this as PMP-sim or *S*_*j*_. A high value of *S*_*j*_, indicates that the CpG location is methylated in normal somatic cells and unmethylated in sperm and cancer cells.

### CTA and CTX gene promoters have prototypical methylation patterns

**Figure 2:**
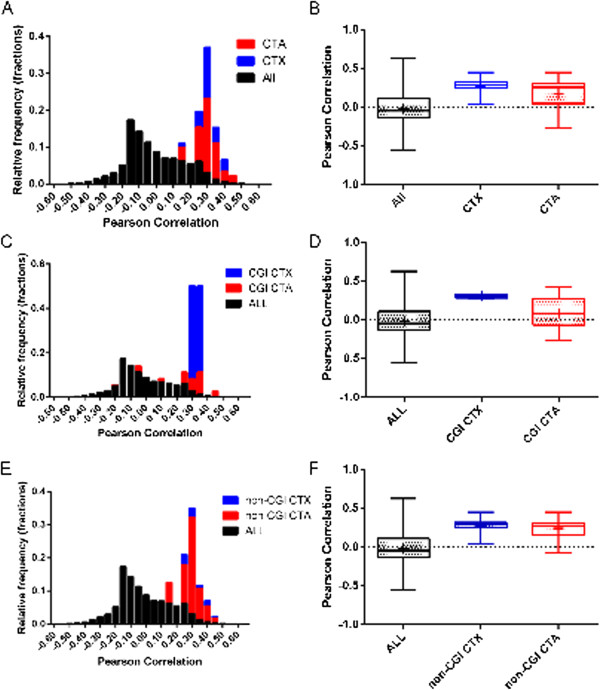
**Pearson correlation values for the CTX and CTA genes are significantly high. **(**A-B**) Pearson correlation distribution in three groups: all the loci (*n* = 27578)*, *CTX loci (*n* = 47), and CTA loci (*n* = 92). A box and whisker plot comparing the correlation values among the three loci groups is shown in (**B**). The plot shows the median, the mean (crosses inside the boxes), 25^th ^percentile (bottom line of the box), 75^th ^percentile (top line of the box), and minimum and maximum values as whiskers. (**C-D**) Pearson correlation distribution in three groups: all the loci (n = 27578), CTX loci in CGI regions (n = 4), and CTA loci in CGI regions (n = 36). (**E-F**) Pearson correlation distribution in three groups: all the loci (n = 27578), CTX loci in non-CGI regions (n = 43), and CTA loci in non-CGI regions (n = 56).

We computed the PMP-sim for all 27578 loci of which 92 correspond to non-X CTA genes (hereon referred to as CTA) and 47 correspond to CTX genes. Overall, the average PMP-sim values was -0.014 ± 0.17, while for the CTA and the CTX genes the average PMP-sim was 0.18 ± 0.17 and 0.27 ± 0.087 respectively (Figure 2). The difference between CTA and all genes was highly significant with Mann–Whitney *U* test *p*-value = 1.47E-21, and likewise for CTX versus all genes with *p*-value = 1.16E-23. Furthermore, the difference between CTX and CTA was also significant (*p*-value = 0.002). We have provided a heatmap (Additional file 2: Figure S1) which clearly shows the prototypical methylation patterns of CTA and CTX genes across 501 samples. Furthermore, we have also demonstrated that several well-known CTAs (MAGE, XAGE, PAGE, and GAGE families) follow the prototypical pattern closely (Additional file 3: Figure S2). All four families had significantly high PMP-sim values: MAGE family (*n* = 19; PMP-sim = 0.27 ± 0.092), XAGE family (*n* = 2; PMP-sim = 0.22 ± 0.045), PAGE family (*n* = 4; PMP-sim = 0.22 ± 0.052), and GAGE family (*n* = 2; PMP-sim = 0.30 ± 0.038). Our conclusion does not change when we constructed the PMP vector by assigning the value of 0 to sperm and cancer cells and the value of 1 to normal somatic cells.

In genome-wide profiling of gene expression and other studies such as DNA methylation, laboratory-specific biases are a significant concern. A visual inspection of the methylation profiles organized by GEO series indicated such a bias. To ensure that our conclusion regarding a greater PMP-sim value for CTA and CTX is not simply because of this bias, we performed the following control. For each CG locus, within each GEO series (Table 1), we randomly permuted the samples. This has the effect of randomizing the normal/tumor identity of the sample while preserving the lab-specific (series-specific) biases. If our observed results above are primarily due to laboratory-specific biases then we would expect the PMP-sim values to be largely preserved. We found this not to be the case. While overall, the PMP-sim did not change significantly (going from 0.014 ± 0.17 for original data to 0.01 ± 0.10 for the permuted data), the PMP-sim for CTA loci was significantly reduced from 0.18 ± 0.17 to 0.03 ± 0.11and that for the CTX genes significantly reduced from 0.27 ± 0.09 to 0.06 ± 0.10. Thus, we conclude that our observed elevated PMP-sim for CTA/CTX loci is not simply due to laboratory-specific biases.

Next, to assess the robustness of our findings above, of the 501 total samples, we randomly sampled 70 samples: 10 normal breast samples (GSE26990), 10 breast cancer samples (GSE26990), 10 normal prostate samples (GSE26126), 10 prostate cancer samples (GSE26126), 10 normal sperm samples (GSE26974), 10 normal colorectal samples (5 from GSE17648 and 5 from GSE25062), and 10 colorectal cancer samples (5 from GSE17648 and 5 from GSE25062). Using these randomly chosen 70 samples instead of 501 samples as above, we computed the PMP-sim for all 27578 loci again and observed high PMP-sim values for the CTA and CTX genes. The results were highly consistent with those obtained when using all 501 samples. The average PMP-sim value across all the loci decreased slightly (going from 0.014 ± 0.17 for the original data to -0.003 ± 0.21 for the re-sampled data). The average PMP-sim values for the CTA and CTX genes were 0.18 ± 0.18 (0.18 ± 0.17 for the original data) and 0.25 ± 0.09 (0.27 ± 0.09 for the original data), respectively. Thus, the results support the robustness of the greater PMP-sim observed for the CTA/CTX loci.

We then partitioned the 27578 promoter CG dinucleotides into 16561 that resided within a CpG island (CGI) and the rest 11017 that did not (non-CGI). Corresponding CGI and non-CGI, counts for CTA genes were 36 and 56 and those for CTX were 4, and 43, respectively. We repeated the above analyses separately for CGI and non-CGI promoters and the results were similarly significant (Figure 2): all genes vs. CGI CTA (Mann–Whitney *U* test *p*-value = 0.0048), all genes vs. CGI CTX (*p*-value = 0.0011), all genes vs. non-CGI CTA (*p*-value = 2.00E-23), and all genes vs. non-CGI CTX (*p*-value = 2.34E-21). Thus, our results suggest that CTA promoters largely follow a PMP across various tissues in both CGI and non-CGI promoters. However, the number of CGI CTX loci was very small, even though the tests showed statistical significance.

### Promoters that follow prototypical methylation pattern are clustered on the genome

**Figure 3:**
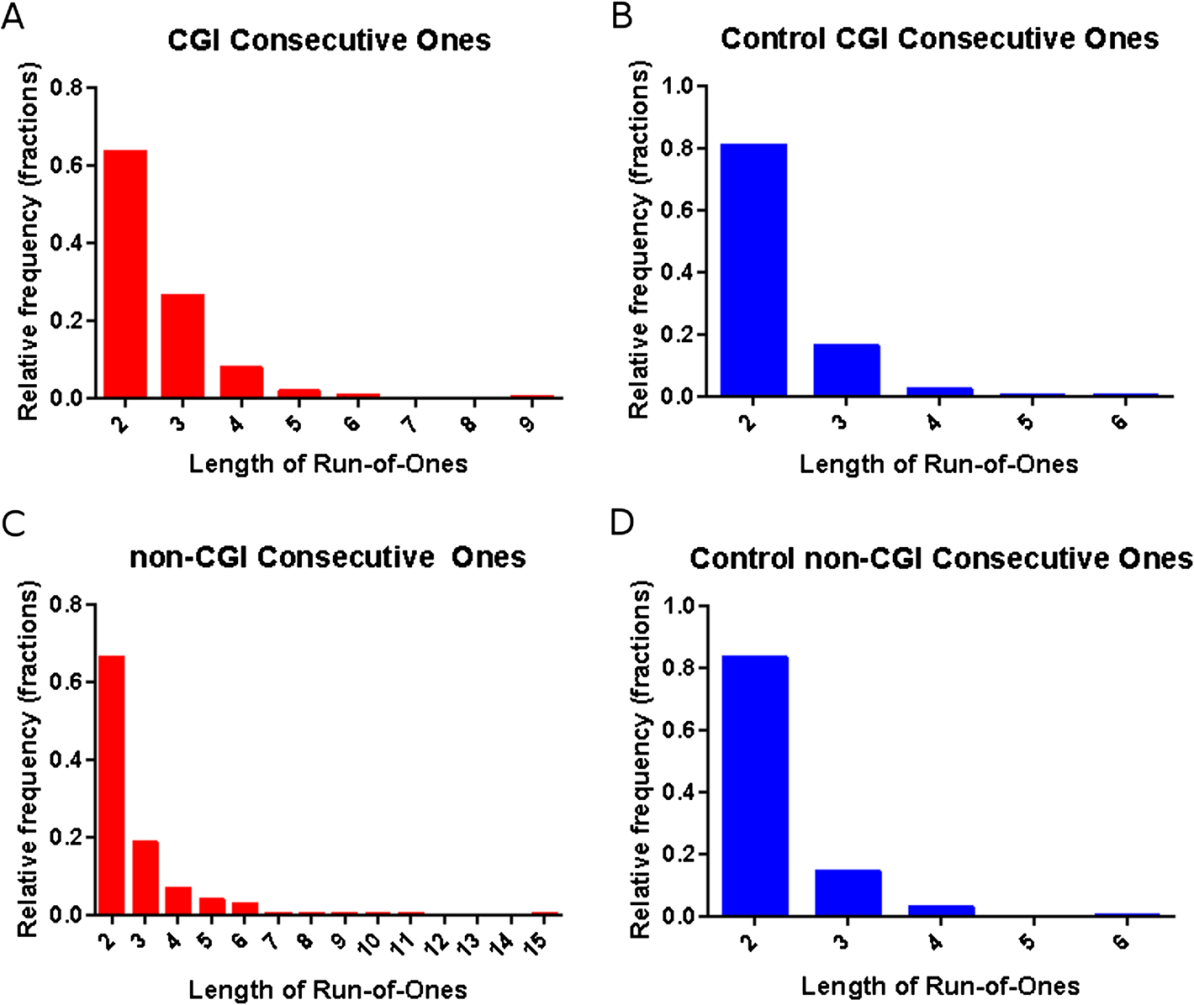
**PMP run lengths for CGIs and non-CGIs were higher than the random control.** Distribution of runs with length of two or greater are shown for (**A**) CGIs, (**B**) randomized CGIs as a control group for (**A**), (**C**) non-CGIs, and (**D**) randomized non-CGIs as a control group for (**C**).

To further understand the mechanism underlying the PMP, we next tested whether the CG dinucleotides that follow PMP, i.e. have high PMP-sim, are clustered on the genome. We identified CG dinucleotides with PMP-sim in top 20^th^ percentile; we refer to this set as high-PMP. We constructed a binary vector of length 27578 corresponding to all CG dinucleotides sorted by their genomic locations. We assigned a ‘1’ at locations corresponding to high-PMP CGs and ‘0’ to the rest. In this binary vector, a *run* was defined as consecutive ‘1’s and the length of a run as the number of consecutive ones in the vector. We identified the runs and their lengths separately for CGIs and non-CGIs promoters. Long runs are suggestive of genomic clustering of PMP promoters. As a control we randomly permuted the binary vector. As shown in Figure 3, we found that for both CGIs and non-CGI promoters the run lengths were significantly higher than that for the corresponding controls (Mann–Whitney *U* test *p*-value = 0.0001 for CGIs and 1.47E-12 for non-CGIs). The average run length for CGIs was 2.348 ± 0.722 (range 1 – 9), and 1.241 ± 0.533 (range 1 – 5) in permuted control. The average run length for non-CGIs was slightly higher at 2.669 ± 1.358 (range 1 – 15) and the corresponding control had run lengths 1.254 ± 0.556 (range 1 – 4). CGIs had 633 runs (26%) with length of two or greater out of 2460 runs. Non-CGIs had 429 runs (28%) with length of two or greater out of 1488 runs. These results, summarized in Figure 3, suggest that promoters with PMP are clustered on the genome for both CGI and non-CGI promoters.

### CTA and CTX gene promoters reside largely within PMP runs

Next, we assessed the extent to which CTA and CTX genes reside within runs of promoters with PMP. As shown in Table 2 for both CGIs and non-CGIs, the fractions of CTA and CTX genes residing within runs were significantly higher than their control groups (binary vectors from run-of-ones analysis permuted 100 times). All comparisons using z-score statistics yielded *p*-value < 4.72E-12. It is also interesting to note that the fractions of CTA and CTX loci that are part of a run (referred to as CTA-F and CTX-F in Table 2) from non-CGIs were far greater than those from CGIs. In other words, more CTA and CTX genes from non-CGIs comprised the runs of length two or greater.

**Table 2:** Fractions of CTA and CTX genes in CGIs and non-CGIs that are in runs with length of two or greater

**CGIs**			
Original Binary Vector		Permuted Binary Vector (avg. ± sd.)	
CTA-F	CTX-F	CTA-F	CTX-F
0.250 (9 out of 36)	1 (4 out of 4)	0.013 ± 0.0248	0.0175 ± 0.0815
**Non-CGIs**			
Original Binary Vector		Permuted Binary Vector	
CTA-F	CTX-F	CTA-F	CTX-F
0.786 (44 out of 56)	0.884 (38 out of 43)	0.226 ± 0.081	0.223 ± 0.077

### CTCF binding sites demarcate PMP from non-PMP regions

CCCTC-binding factor (CTCF) is a multifunctional protein best known for its role as an insulator of epigenomic marks [38,39]. Thus, we asked whether the presence of CTCF binding sites has any bearing on PMP at consecutive CG loci intervened by CTCF binding. A previous study comparing CTCF binding in multiple cell types had shown that a large fraction of CTCF binding events are conserved across cell types [40]. Thus, in our analysis we only included 7428 CTCF sites that were common to 4 cell types, namely CD4+ T cell, IMR90, Hela, and Jurkat. Details of individual datasets and extraction method are provided in [40]. For each CTCF binding site we assessed its insulator tendency as the absolute difference in average PMP-sim between 3 CG loci to the 5’ of the CTCF site and 3 CG loci to the 3’ of the CTCF site (see Methods); we refer to this value as delta-PMP. As a control for CTCF sites, we chose random loci in the genome.

**Figure 4:**
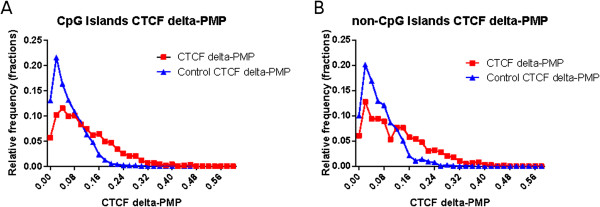
**Delta-PMP values for the CTCF sites in CGIs and non-CGIs were higher than the random control. **(**A**) delta-PMP distribution for the CTCF sites in CGIs (red) and its control (blue). (**B**) delta-PMP distribution for the CTCF sites in non-CGIs (red) and its control (blue).

Interestingly, we found that for both CGIs and non-CGIs promoters, the delta-PMP for CTCF sites were significantly higher than the random control (Figure 4). The average delta-PMP for CGIs was 0.12 ± 0.09, while the random delta-PMP was 0.062 ± 0.05 (Mann–Whitney *U* test *p*-value = 1.38E-217). A similar trend was observed in non-CGIs; the mean CTCF delta was 0.12 ± 0.09, while the mean random delta was 0.07 ± 0.05 (Mann–Whitney *U* test *p*-value =7.59E-52). Moreover, the delta-PMP distribution of CGIs and non-CGIs was statistically indistinguishable. Further, when we tested whether the CTAs or runs of CTAs reside near a CTCF site relative to random expectation, we found this not to be the case, suggesting that potential involvement of CTCF sites in demarcating PMP regions is not relevant to CTAs and is instead a general phenomenon.

### Promoters with PMP intersect with Lamin Attachment Domains (LADs)

**Figure 5:**
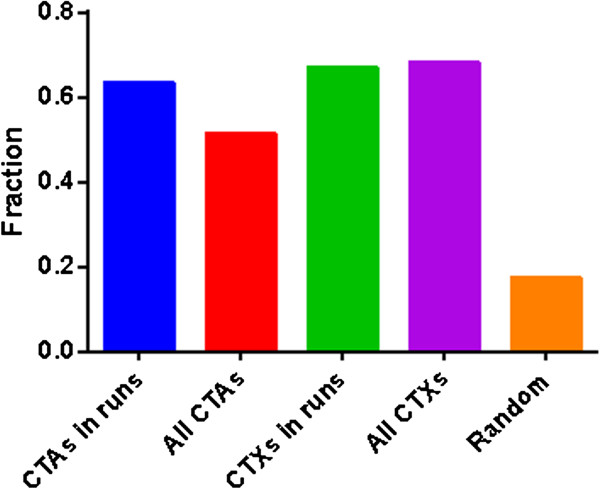
**CTA and CTX loci intersect with LAD regions more frequently. **Out of 92 CTA loci, 47 (51.09%) resided within a LAD (red bar); Out of 19 CTA loci in PMP runs, 12 (64.16%) of them resided within a LAD (blue bar); Out of 47 CTX loci, 32 (68.09%) resided within a LAD (purple bar); Out of 12 CTX loci in PMP runs, 8 (66.67%) of them resided within a LAD (green bar). Out of 1000 loci randomly selected, 173 (17.30%) resided within a LAD (orange bar).

Regions of hypomethylation in cancer have previously been shown to significantly intersect with LADs and are thus thought to be critical in organizing the interphase chromosomes [41]. We extracted 1344 LAD loci from [42]. Out of 5397 high-PMP (CGI and non-CGI loci combined) 1389 (25.74%) resided within a LAD, and of the 21558 other CG loci only 3229 (14.98%) resided within a LAD. This difference was statistically highly significant (Fisher’s exact test *p*-value = 8.17E-50). The difference is similarly significant when CGI and non-CGI loci were analyzed separately. This result along with our finding that CTA (and CTX) CG loci have high PMP-sim would suggest a high correlation between CTAs and LADs. This is indeed the case and as shown in Figure 5, the fraction of CTA and CTX loci, both overall, as well as the ones that intersect with high-PMP runs, intersect with LAD regions five- to six-fold more frequently than random expectations: all comparisons using z-score statistics yielded an extremely small *p*-value (almost 0). In addition, we found that the CG loci within LADs had significantly greater PMP-sim values than the CG loci outside LADs (Mann–Whitney *U* test *p*-value = 8.20E-31). Thus, our results suggest that high-PMP runs, and consequently CTA and CTX loci with PMPs largely intersect with LAD regions.

Given that CTCF sites demarcate PMP from non-PMP regions, that PMP regions overlap with LAD, and that LAD were previously shown to be demarcated by CTCF sites [42], we expect that LAD boundaries themselves demarcate PMP from non-PMP regions. Similar to our previous analysis performed on the CTCF binding sites, we assessed the tendency of LAD boundaries to exhibit high difference in PMP-sim for the three CG loci to the 5’ and the three CG loci to the 3’. As expected we observed a trend similar to that observed for CTCF sites. The delta-PMP values for LAD boundary loci were significantly higher than the random control for both CGIs and non-CGIs promoters. The average delta-PMP for LAD loci in CGIs was 0.11 ± 0.089, while the control delta-PMP was 0.063 ± 0.052 (Mann–Whitney *U* test *p*-value = 8.27E-30). The average delta-PMP for LAD loci in non-CGIs was 0.11 ± 0.094, while its control delta-PMP was 0.071 ± 0.055 (*p*-value = 2.43E-7).

## Discussion

Even amongst the so-called tissue-specific genes, the CTAs exhibit a remarkable expression pattern. While typically expressed only in the sperm and repressed in normal somatic tissues, they are aberrantly derepressed in most cancers [1]. However, neither the mechanism nor the functional consequence of this atypical expression pattern is entirely clear for most, if not all, CTAs. While there is evidence to suggest that promoter demethylation might be a major driver of derepression of CTA expression in cancer [3], this mechanism does not appear to be universally applicable to all CTAs [34]. Independent of CTA-related investigations, it has been shown that large genomic regions are hypomethylated in some cancers [41,43]. It is therefore tempting to speculate that CTAs may be swept by the global hypomethylation as bystanders leading to their derepression. Based on a systematic analysis of DNA methylation profiling data from various tissues, our results support this thesis.

We found specific hypomethylation of the CTA and CTX promoters in the testis and cancer cells. More specifically, we observed hypomethylation of MAGE, XAGE, PAGE, and GAGE promoters in cancer samples (Additional file 3: Figure S2) confirming several studies that have reported that the activation of these genes in cancer is strongly correlated with promoter demethylation [23,44,45]. This result, combined with well-established association between DNA methylation and gene silencing suggests methylation as the predominant mechanism for CTA derepression in cancers. Moreover, the loci with PMP including the ones in CTA and CTX promoters, cluster on the genome and are associated with LAD regions. This is consistent with broad regions of hypomethylation in cancers that are also associated with LAD regions [41]. Taken together, these findings suggest that hypomethylation and derepression of CTA and CTX genes in cancers are part of a broader phenomenon and may not depend on a specific mechanism. We also found that the broad tendencies revealed by our analyses are independent of CpG islands. This may suggest a non-specific mechanism underlying methylation-mediated derepression of CTA genes. Furthermore, we also found that CTCF sites are linked to a sudden change in methylation patterns for both CGIs and non-CGI loci. This is consistent with a previous study that found epigenetic silencing of tumor suppressor genes in the absence of CTCF binding [46].

We note that the methylation profiling platform (Illumina HumanMethylation27 BeadChip) used in this study includes only ~1 CpG locus per gene promoter, resulting in a small number loci corresponding to CTA and CTX genes. Furthermore, a single CpG site may not be representative of an entire promoter in all cases. Although Illumina has a newer and denser methylation chip (Illumina HumanMethylation450 BeadChip) which contains more than 450,000 methylation sites, the number of relevant tissues for which such data exists is currently insufficient. In addition, although previous works have illustrated an inverse correlation between promoter methylation and gene expression level, we could not ascertain this for our data because none of the samples included in the study had a corresponding expression data available.

## Conclusions

In conclusion, here we present a systematic analysis based on large number of genome-wide methylation profiles across multiple normal and tumor cells demonstrating that in general, derepression of CTA and CTX genes in cancer may be largely explained by global hypomethylation mediated by disruption of laminar attachment regions. However, it is important to note that, DNA hypomethylation may not be the only mechanism since several CTAs lacking CpG islands are also upregulated in response to DNA hypomethylation [47]. Thus, it is possible that at least for some CTA genes, additional mechanisms for repression and alternative mechanisms for derepression in cancer exist which may involve specific repressive or activating transcription factors. Additional biochemical studies elucidating global methylation changes should yield new insights on regulation of CTA expression paving the way to the development of novel therapeutic modalities for cancer. This is particularly important since epigenetic modulation of CTA expression is emerging as a novel medical modality for cancer immunotherapy [32,48,49].

## Abbreviations

CTA: Cancer/Testis Antigen; CTX: Cancer/Testis X Antigen; CGI: CpG island; nCGI: non-CpG island; PMP: Prototypical methylation pattern; CTCF: CCCTC-binding factor; CTCFL: CTCF-like protein; LAD: Lamina associated domain.

## Competing interests

The authors have no competing interests to declare.

## Authors’ contribution

The study was conceived by P.K. and S.H. All analysis was done by R.K. The manuscript was written jointly by all authors. All authors read and approved the final manuscript.

## Pre-publication history

The pre-publication history for this paper can be accessed here:

http://www.biomedcentral.com/1471-2407/13/144/prepub

## Supplementary Material

Additional file 1: Table S1 The PMP vector for 501 samples is shown in the file. Click here for file

Additional file 2: Figure S1 Heatmap of the methylation levels of CTA, CTX, and non-CTA loci across 501 samples. Heatmap of methylation data shows the prototypical methylation patterns of CTA and CTX loci: high methylation levels (yellow in the heatmap) in normal samples and low methylation levels (green in the heatmap) in cancer and sperm cells. On the other hand, the methylation levels of 150 randomly selected non-CTA loci did not follow the prototypical methylation patterns. Click here for file

Additional file 3: Figure S2 Heatmap of the methylation levels of MAGE, XAGE, PAGE, and GAGE promoter loci across 501 samples. Heatmap of methylation data shows that these four CTX families follow the prototypical methylation patterns: high methylation levels (yellow in the heatmap) in normal samples and low methylation levels (green in the heatmap) in cancer and sperm cells. The average PMP-sim values for the four families are: 0.27 ± 0.092 (MAGE family; n = 19), 0.22 ± 0.045 (XAGE family; n = 2), 0.22 ± 0.052 (PAGE family; n = 4), and 0.30 ± 0.038 (GAGE family; n = 2). The PMP vector is shown in the bottom part of the figure (boxed), and the PMP-sim value for each gene is shown on the right vertical axis. Click here for file
