# Supplementary figures and images for: Derepression of Cancer/Testis Antigens in cancer is associated with distinct patterns of DNA Hypomethylation
Source: BMC Cancer. 2013 Mar 22;13:144. doi: 10.1186/1471-2407-13-144 (PMC3618251; doi:10.1186/1471-2407-13-144)

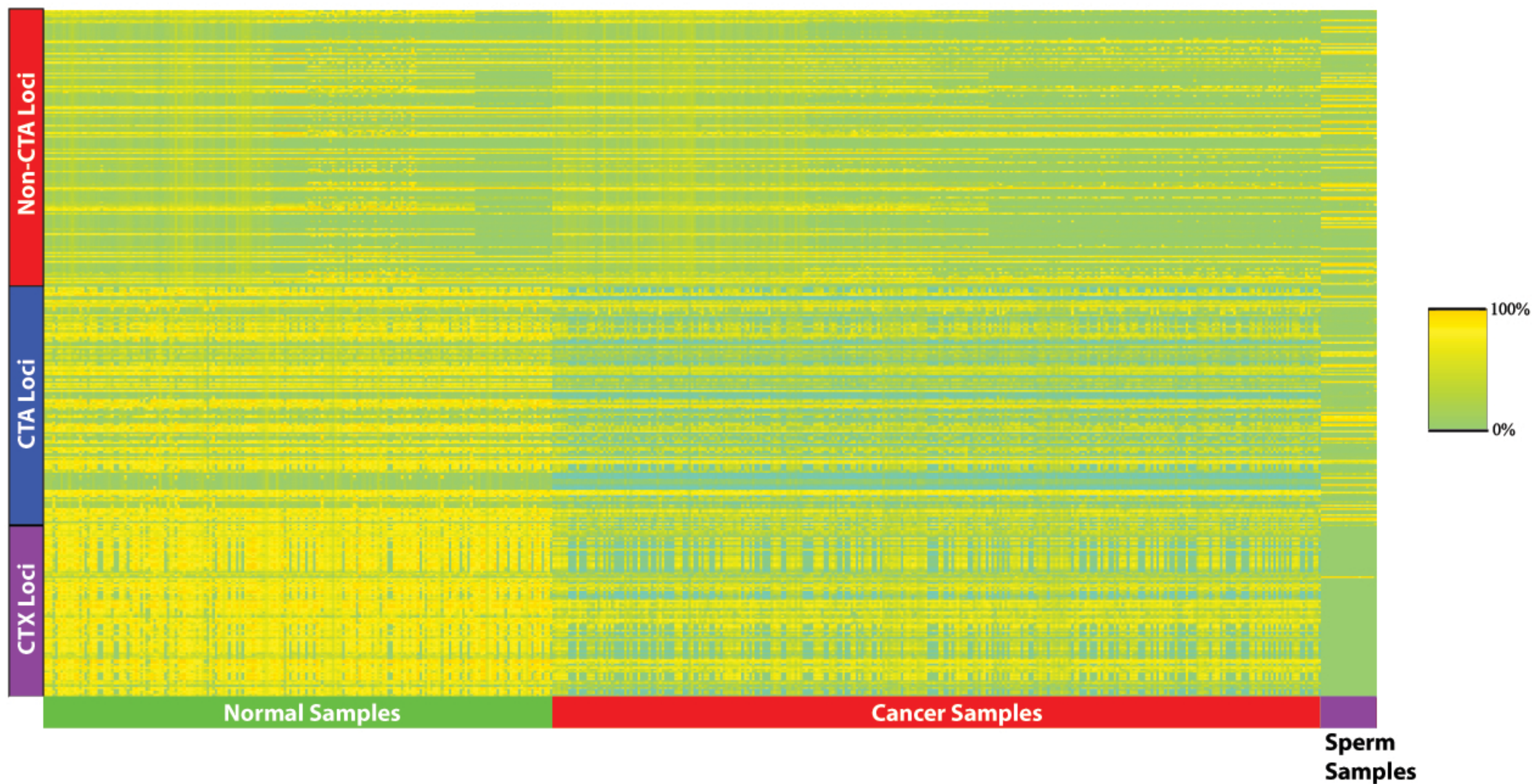

Supplement: Additional file 2: Figure S1 — Heatmap of the methylation levels of CTA, CTX, and non-CTA loci across 501 samples. Heatmap of methylation data shows the prototypical methylation patterns of CTA and CTX loci: high methylation levels (yellow in the heatmap) in normal samples and low methylation levels (green in the heatmap) in cancer and sperm cells. On the other hand, the methylation levels of 150 randomly selected non-CTA loci did not follow the prototypical methylation patterns. [file 1471-2407-13-144-S2.pdf]

Normal Samples

Cancer Samples

Sperm

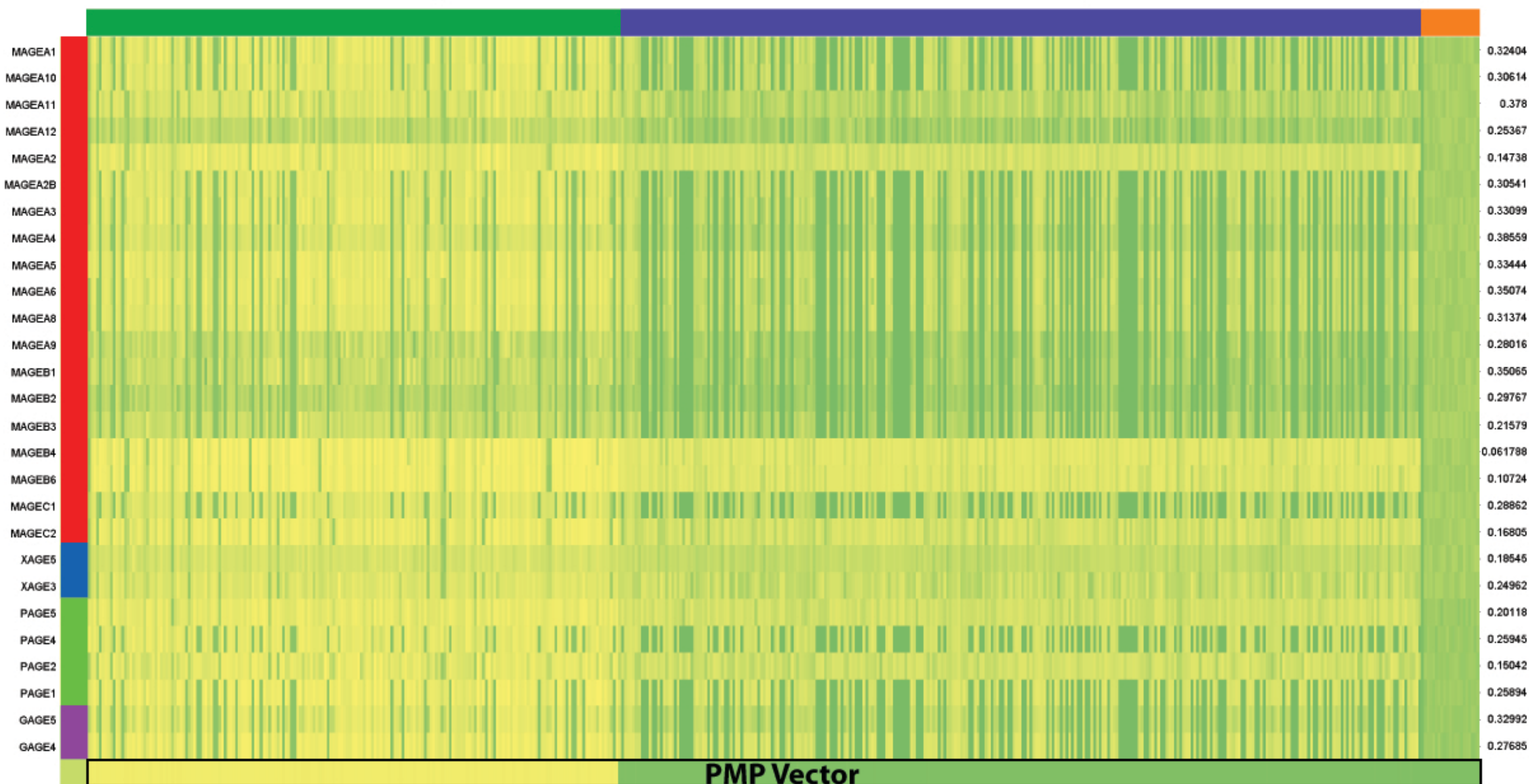

PMP-sim Values

Methylation Level

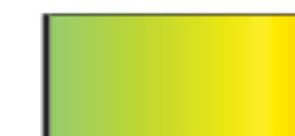

0%

100%

Supplement: Additional file 3: Figure S2 — Heatmap of the methylation levels of MAGE, XAGE, PAGE, and GAGE promoter loci across 501 samples. Heatmap of methylation data shows that these four CTX families follow the prototypical methylation patterns: high methylation levels (yellow in the heatmap) in normal samples and low methylation levels (green in the heatmap) in cancer and sperm cells. The average PMP-sim values for the four families are: 0.27 ± 0.092 (MAGE family; n = 19), 0.22 ± 0.045 (XAGE family; n = 2), 0.22 ± 0.052 (PAGE family; n = 4), and 0.30 ± 0.038 (GAGE family; n = 2). The PMP vector is shown in the bottom part of the figure (boxed), and the PMP-sim value for each gene is shown on the right vertical axis. [file 1471-2407-13-144-S3.pdf]
